# A 44‐year‐old man with esophageal ulcer

**DOI:** 10.1002/ccr3.6044

**Published:** 2022-07-18

**Authors:** Delvise T. Fogwe, Matthew T. Ho, Aditya S. Shah

**Affiliations:** ^1^ Department of Internal Medicine Mayo Clinic Rochester Minnesota USA; ^2^ Department of Infectious Diseases Mayo Clinic Rochester Minnesota USA

**Keywords:** cytomegalovirus, esophagitis, immunocompromised, ulcer

## Abstract

This report presents a classic case of CMV esophagitis, which may be puzzling to distinguish from other infectious esophageal lesions. Giant (>1 cm) and deep esophageal lesions in immunocompromised patients may suggest CMV esophagitis. A biopsy with immunostaining is needed to confirm the diagnosis.

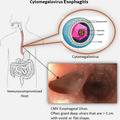

## 1 QUESTION

**FIGURE 1:**
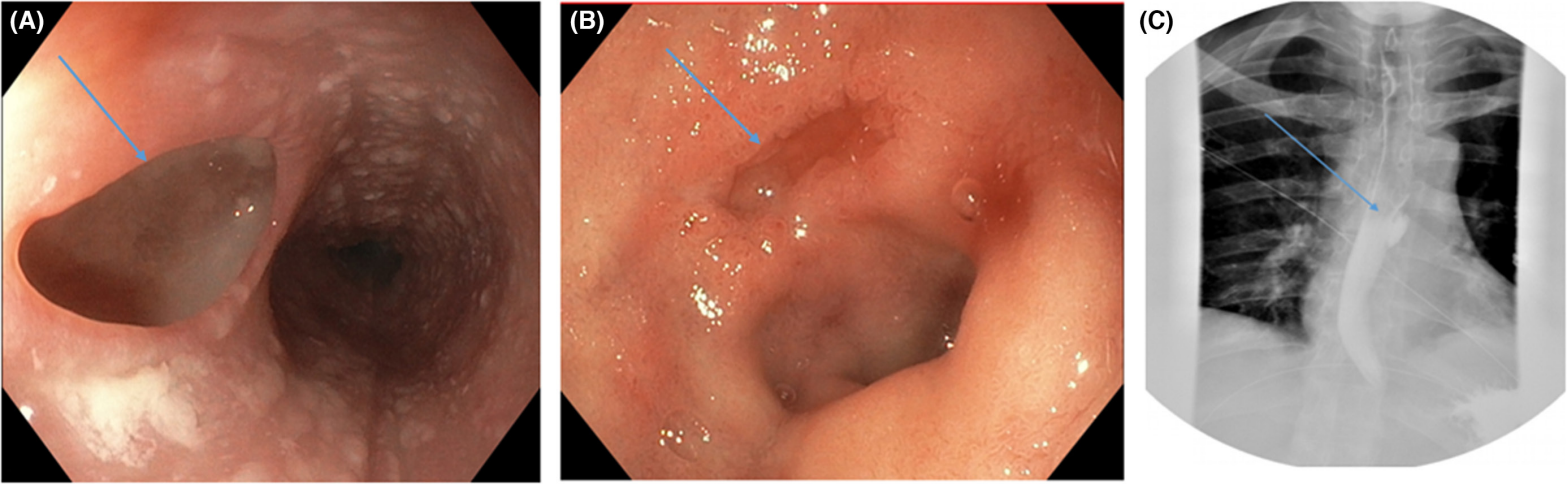
(A) Deep esophageal ulcer. (B) Gastric ulcer. (C) Barium swallow study

A 44‐year‐old man was admitted for acute gastrointestinal bleed, intermittent esophageal dysphagia, and odynophagia. His past medical history was significant for dermatomyositis, for which he was on mycophenolate but had previously received prednisone and methotrexate. The treatment for his dermatomyositis had been complicated by pancytopenia with hemoglobin 6.8 (13.2–16.6 g/dl), platelet 73 (135–317 × 10[9]g/L), and white blood cells 1.9(3.4–9.6 × 10[9]g/L). He underwent esophagogastroduodenoscopy (EGD), which showed a deep esophageal ulcer on the background of diffuse white plaques in the middle third of the esophagus and a non‐bleeding large cratered clean‐based gastric antral ulcer (Figure 1A,B). Barium swallow showed a sac‐like structure consistent with the ulcer without contrast leakage to suggest a fistula (Figure 1C). What is the most likely diagnosis?

## 2 ANSWER

This is a classic presentation of cytomegalovirus (CMV) esophageal ulcer. The biopsy of the ulcer revealed positive CMV immunostaining, with negative immunostaining for Herpes simplex virus and fungus. His quantitative CMV DNA was 20,400 IU/ml (reference range undetected), and his HIV test was negative. CMV predominantly causes opportunistic infection in immunosuppressed patients. It mainly causes gastrointestinal symptoms, with about 12.9% of patients presenting with esophageal disease.^1^ Unlike other infectious esophageal ulcers, CMV tends to cause one or more giant (>1 cm) ulcers that may be flat, ovoid, or diamond‐shaped.^2^

## AUTHOR CONTRIBUTIONS

Delvise Fogwe, first author, wrote and edited the case report. Matthew Ho, second author, assisted with acquiring images and putting images together. Aditya S. Shah, third author, provided overall mentorship and guidance on the direction of the paper.

## CONFLICT OF INTEREST

None.

### CONSENT

Written informed consent was obtained from the patient to publish this report in accordance with the journal's patient consent policy.

## Data Availability

The authors confirm that the data supporting the findings of this study are available within the article [and/or] its supplementary materials.
